# The preventive effect of *Gastrodia elata* Blume extract on vancomycin-induced acute kidney injury in rats

**DOI:** 10.1186/s42826-024-00200-y

**Published:** 2024-04-08

**Authors:** Yeon Su Lee, Yu Rim Park, Hyo Bin Lee, Hye Joon Park, Ha Eun Lee, Geon A Kim, Sang-Hoon Kim, Jae-Ho Shin

**Affiliations:** 1 Department of Senior Healthcare, Eulji University, Uijeongbu, 11549 Korea; 2 Department of Biomedical Laboratory Science, Eulji University, Seongnam, 13135 Korea; 3 Department of Biomedical Laboratory Science, Eulji University, Uijeongbu, 11759 Korea; 4 Department of Internal Medicine, Eulji Hospital, Seoul, 01830 Korea; 5 Eulji Medi-Bio Research Institute, Eulji University School of Medicine, Seoul, 01830 Korea

**Keywords:** *Gastrodia elata* Blume, Acute kidney injury, Preventive effect, Anti-inflammation, Anti-oxidation

## Abstract

**Background:**

*Gastrodia elata* Blume (GEB), a traditional medicinal herb, has been reported to have pharmacological effect including protection against liver, neuron and kidney toxicity. However, explanation of its underlying mechanisms remains a great challenge. This study investigated the protective effects of GEB extract on vancomycin (VAN)-induced nephrotoxicity in rats and underlying mechanisms with emphasis on the anti-oxidative stress, anti-inflammation and anti-apoptosis. The male Sprague-Dawley rats were randomly divided three groups: control (CON) group, VAN group and GEB group with duration of 14 days.

**Results:**

The kidney weight and the serum levels of blood urea nitrogen and creatinine in the GEB group were lower than the VAN group. Histological analysis using hematoxylin & eosin and periodic acid Schiff staining revealed pathological changes of the VAN group. Immunohistochemical analysis revealed that the expression levels of N-acetyl-D-glucosaminidase, myeloperoxidase and tumor necrosis factor-alpha in the GEB group were decreased when compared with the VAN group. The number of terminal deoxynucleotidyl transferase-mediated dUTP nick end labeling-positive cells, phosphohistone and malondialdehyde levels were lower in the GEB group than VAN group. The levels of total glutathione in the GEB group were higher than the VAN group.

**Conclusions:**

The findings of this study suggested that GEB extract prevents VAN-induced renal tissue damage through anti-oxidation, anti-inflammation and anti-apoptosis.

**Supplementary Information:**

The online version contains supplementary material available at 10.1186/s42826-024-00200-y.

## Background

Acute kidney injury (AKI) is a pathological condition characterized by rapid deterioration of renal functions that can result in health complications through the accumulation of body fluids and waste products [1, 2]. Additionally, AKI can result in death if left untreated. The pathological characteristics of AKI are decreased glomerular filtration rate and increased serum levels of blood urea nitrogen (BUN) and creatinine (CREA) [1]. The drugs such as antibiotics are one of the major etiological factors for AKI [1]. In this study, vancomycin hydrochloride (VAN), an antibiotic, was used to induce AKI.

VAN is a glycopeptide antibiotic that is used to treat bacterial infections, especially methicillin-resistant *Staphylococcus aureus* infections [3]. It also has the same chemical structure as Supplementary Figure 1. However, VAN is associated with several side effects, including AKI [4–6]. Some recent studies have reported that oxidative stress and inflammation are potential mediators of VAN-induced AKI [4, 5, 7]. The renal accumulation of VAN promotes oxidative stress and inflammation, which consequently enhance the levels of reactive oxygen species (ROS), such as superoxide radical and hydrogen peroxide [6, 8]. ROS promote nuclear factor-κB (NF-κB) activation, which enhances cell proliferation and apoptosis and promotes the secretion of pro-inflammatory cytokines, including tumor necrosis factor-alpha (TNF-α) [9]. The enhanced levels of ROS promote renal cell damage and decrease renal function by promoting inflammation through the upregulation of inflammation-related factors [3, 7, 8, 10].

*Gastrodia elata* Blume (GEB) has been used to treat headaches, epilepsy, dizziness, rheumatism, neuralgia, cramps, high blood pressure, and inflammation in traditional Chinese medicine [11, 12]. Previous studies have reported that GEB inhibits ROS production, inflammation, and lipid peroxidation [13, 14]. The main bioactive component of GEB is p-hydroxybenzyl alcohol (HBA), which exhibits anti-inflammatory activity through the inhibition of ROS production [14, 15]. Therefore, we investigated it as it was believed to have a preventive effect against increased oxidative stress and inflammation caused by AKI.

This study investigated the preventive effects of GEB against VAN-induced AKI in rats and the underlying mechanisms.

## Methods

### Preparation of GEB extract

The aqueous extract of GEB was provided by MJ Health Foods Co. (Muju, Jeollabuk-do, Korea). The roots of GEB were subjected to hot air-drying. The dried roots of GEB were cut into small pieces and extracted with 10 times the volume of distilled water (DW) at 110 ℃ for more than 20 h. The insoluble material was removed using 25-µm and 5-µm filters and the filtrate was concentrated under vacuum. The concentrate was lyophilized. The lyophilized GEB extract was sterilized at 121 ℃ for 15 min. Next, DW was added to the extract and the extract was concentrated again under reduced pressure. After then, 1mL of GEB extract and 50% methanol to a 15mL tube and mix well. The mixed sample is subjected to ultrasasonic extraction (USE, ultrasonification extraction) for 30 minutes with an ultrasonic extractor. Then, after centrifugation at 1,000 rpm for 10 minutes with a centrifuge, the supernatant except for the settled solid was filtered with a 0.45μm syringe filter. And High Performance Liquid Chromatography (HPLC) was used to analyze GEB extract, and the conditions are shown in Table 1.

**Table 1:** HPLC-DAD analysis conditions for GEB extract analysis

**LC**		**Agilent 1100 series**						
**Column**		**Capcellpak C18 UG1200 (4.6x250mm, 5μm)**						
**Mobile phase**		**A: Water: ACN = 99:1** **B: ACN**						
**Gradient mode**		**Time (min)**	0	15	20	25	25.1	30
		B (%)	0	0	90	90	90	0
Flow		1.2 mL/min						
Injection volume		10μL						
Detector	DAD Detector	270nm						

We confirmed the amount of HBA, an active ingredient, present in the GEB extract used in this experiment (Table 2).

**Table 2:** HBA content of GEB extract confirmed using HPLC-DAD

	**Condition**	**Raw material**	**After filtration**	**After condensation**
**4-HBA**	Contents (mg/g)	1.671	0.041	0.294

### Chemicals and reagents

VAN, which was purchased from CJ healthcare (Seoul, Korea), was dissolved in sterilized saline. Primary antibodies against n-acetyl-β-D-glucosaminidase (NAG; ab214671, Abcam, Cambridge, UK), myeloperoxidase (MPO; ab208670, Abcam), TNF-α (ab66579, Abcam), and ABC-horseradish peroxidase (HRP) (peroxidase, Rabbit IgG) kit (PK6101, Vector Laboratories, Burlingame, CA, USA) were used for immunohistochemical and western blotting analyses. To examine apoptosis in the renal sections, terminal deoxynucleotidyl transferase dUTP nick end labeling (TUNEL) staining was performed using *in situ* cell death detection kit (POD, cat #684 817 910, Roche, Basel, Switzerland). The glutathione levels were examined using the total glutathione assay kit (STA-312, Cell Biolabs, San Diego, CA, USA). The levels of malondialdehyde (MDA), which indicate the levels of lipid peroxidation, were examined using the OxiSelect™ thiobarbituric acid reactive substances assay kit (STA-330, Cell Biolabs).

### Animals

Male Sprague-Dawley rats (aged 5 weeks with a bodyweight (BW of 180–200 g) were purchased from Samtako Bio (Osan, Gyeonggi-do, Korea). The animals had free access to food (Purina, Seongnam, Gyeonggi-do, Korea) and tap water and were maintained under the following conditions: temperature, 22 ± 2℃; humidity, 50 ± 10%; circadian cycle, 12-h light/dark cycle. The BW and food and water intake were measured twice a week during the experimental period. The experimental procedure was approved by the Institutional Animal Care and Use Committee of Eulji University (EUIACUC 17-01).

### Experimental design

The animals, which were allowed to adapt to the standard housing conditions for 7 days, were randomly divided into the following three groups: control (CON) group, orally administered DW (10 mL/kg BW); VAN group, orally administered DW (10 mL/kg BW); GEB group, orally administered GEB extract (10 mL/kg/BW). The treatment period for all groups was 14 days. During the last 3 days of the 14-day treatment period, the VAN and GEB groups were intraperitoneally administered VAN (400 mg/kg BW) after oral administration, whereas the CON group was intraperitoneally administered physiological saline (5 mL/kg BW).

Oral administration and/or intraperitoneal injection were performed in the morning once a day during the experimental period. At 24 h after the last VAN injection, the rats were allowed to fast overnight and anesthetized using isoflurane (Halocarbon, Georgia, USA) (Supplementary Figure 2).

The blood sample was collected from the abdominal aorta of the rat into a serum-separating tube (BD, New Jersey, USA). Next, the blood samples were centrifuged at 3,000 rpm and 4℃ for 10 min to obtain the serum. The serum was stored at −80 ℃. Further, the kidney was excised and weighed immediately. The relative kidney weight was

Calculated as the ratio of kidney weight to BW and was expressed as a percentage. The left kidney was fixed in a 10% formalin solution for histological analysis, whereas the right kidney was frozen in liquid nitrogen and stored at −80 ℃ for biochemical analysis.

### Measurements of serum BUN and CREA in serum

To evaluate kidney function, the serum levels of BUN and CREA were measured at the Green Cross-reference lab (Gyeonggi-do, Korea). BUN was measured using ultraviolet spectrophotometry with UREAL kit (Roche) and Cobas 8000 (c702, Roche). CREA was measured using the colorimetric method with CREJ2 kit (Roche) and Cobas 8000 (c702, Roche).

### Histological analysis

The fixed renal tissue was embedded in paraffin and sectioned into 4-μm thick sections. The sections were subjected to hematoxylin and eosin (H&E) and periodic acid Schiff (PAS) staining and observed under a light microscope (Olympus, Tokyo, Japan).

Renal damage in the H&E-stained sections was scored based on the modified method of Cetin H et al (2007) in a blinded manner. The degree of renal damage was assessed by scoring tubular dilation, tubular epithelial desquamation, tubular vacuolation, interstitial inflammation, interstitial edema, and tubular cast as follows: 0=no damage; 1=mild damage; 2=moderate damage; 3=severe damage (Table 3).

**Table 3:** Scoring methods for histological observation

**Histopathological parameter**	**Grade**
Tubular dilation Tubular epithelial desquamation Tubular vacuolation Interstitial inflammation Interstitial edema Cast	0 = no damage 1 = mild damage 2 = moderate damage 3 = severe damage

PAS staining was performed to examine the changes in the tubular brush border and glomerular basement membrane.

### Immunohistochemical analysis

Immunohistochemical analysis was performed using anti-NAG to evaluate the renal tubular injury and anti-MPO and anti-TNF-α antibodies to evaluate inflammatory infiltrated that promotes renal injury. To perform immunohistochemical analysis with anti-NAG antibodies, the sections were boiled in 1X citrate buffer (pH 6.4). Additionally, the sections were incubated at 37℃ with proteinase K (Invitrogen, California, USA) and 0.1% trypsin (Sigma-Aldrich, Missouri, USA) for 30 min for antigen retrieval to perform immunohistochemical analysis with anti-MPO and anti-TNF-α antibodies. All sections were incubated with anti-NAG (1:2,000), anti-MPO (1:4,000), and anti-TNF-α (1:200) antibodies at 4℃ overnight. The sections were then incubated with the secondary antibody (biotinylated goat anti-rabbit IgG) at room temperature for 30 min, followed by incubation with avidin-biotin reagent and 3,3-diaminobenzidine tetrahydrochloride (DAB, Sigma-Aldrich). Further, the sections were observed under a light microscope (Olympus) for histological evaluation.

### Terminal deoxynucleotidyl transferase dUTP nick end labeling (TUNEL) assay in the kidney

TUNEL assay was performed to examine DNA fragmentation. This assay labels the 3′-hydroxyl termini of the double-strand DNA breaks generated during apoptosis.

The renal tissues were sectioned on silane-coated slides and deparaffinized. The sections were incubated at 37℃ with 0.1% trypsin (in 1X PBS) for 30 min for antigen retrieval and inhibited endogenous peroxidase activity. The label solution was incubated with the enzyme solution at 37℃ for 1 h. The sections were incubated with peroxidase (POD) at 37℃ for 30 min. Next, the sections were incubated with DAB and counterstained with Mayer hematoxylin. Five random fields in the sections were observed under a light microscope (Olympus) at 400X magnification to identify the TUNEL-positive cells. The number of TUNEL-positive cells was divided by the total number of cells and expressed as previously described.

### Measurement of antioxidant enzymes in the kidney

The renal tissue (100 mg) was homogenized in 1 mL of 5% metaphosphoric acid using a homogenizer. The homogenate was centrifuged at 12,000 rpm and 4 °C for 15 min. The supernatant was used for the assay or immediately stored at −80 ℃. Total glutathione (GSH) levels in the supernatants were measured using the total glutathione assay kit (Cell Biolabs).

### Measurement of oxidative stress in the kidney

The renal tissue (100 mg) was homogenized in 1 mL of 1X PBS using a homogenizer. The homogenate was incubated with 10 µL of 100X butylated hydroxytoluene solution to prevent further oxidation. The mixture was centrifuged at 10,000 *g* and 4 °C for 5 min. The supernatant was used for the assay or immediately stored at −80 ℃.

The MDA levels in the supernatant were measured using OxiSelect™ thiobarbituric acid reactive substances assay kit (Cell Biolabs).

### Western blotting analysis

Equal amounts of protein (13 μg) were subjected to SDS-PAGE using a 10% gel for analyzing the levels of NAG (82kD) β-actin or a 12% gel for analyzing the levels of MPO (59kD). The resolved proteins were transferred to a polyvinylidene fluoride membrane. The membrane was blocked with 5% skim milk for 1 h and washed with Tris-buffered saline containing 0.1% Tween-20 (TBS-T, Sigma-Aldrich). Next, the membrane was incubated with the following primary antibodies at 4 °C overnight: anti-NAG (1:1,000), anti-MPO (1:1,000), and anti-actin (1:150,000, Abcam) antibodies. The membrane was washed with TBS-T and incubated with HRP-conjugated anti-rabbit IgG (1:2,000, Pierce, Rockford, IL, USA) and anti-mouse IgG (1:3,000, Pierce) for 1 h. Immunoreactivity was detected using an X-ray film.

### Statistical analysis

The means of multiple groups were compared using one-way analysis of variance, followed by least significant difference post-hoc analysis with SPSS (version 10.0; SPSS Inc., Chicago, USA). All data are expressed as mean ± standard error. The differences were considered significant at *p* < 0.01 or 0.05.

## Results

### Effects of GEB on body weight

**Fig. 1:**
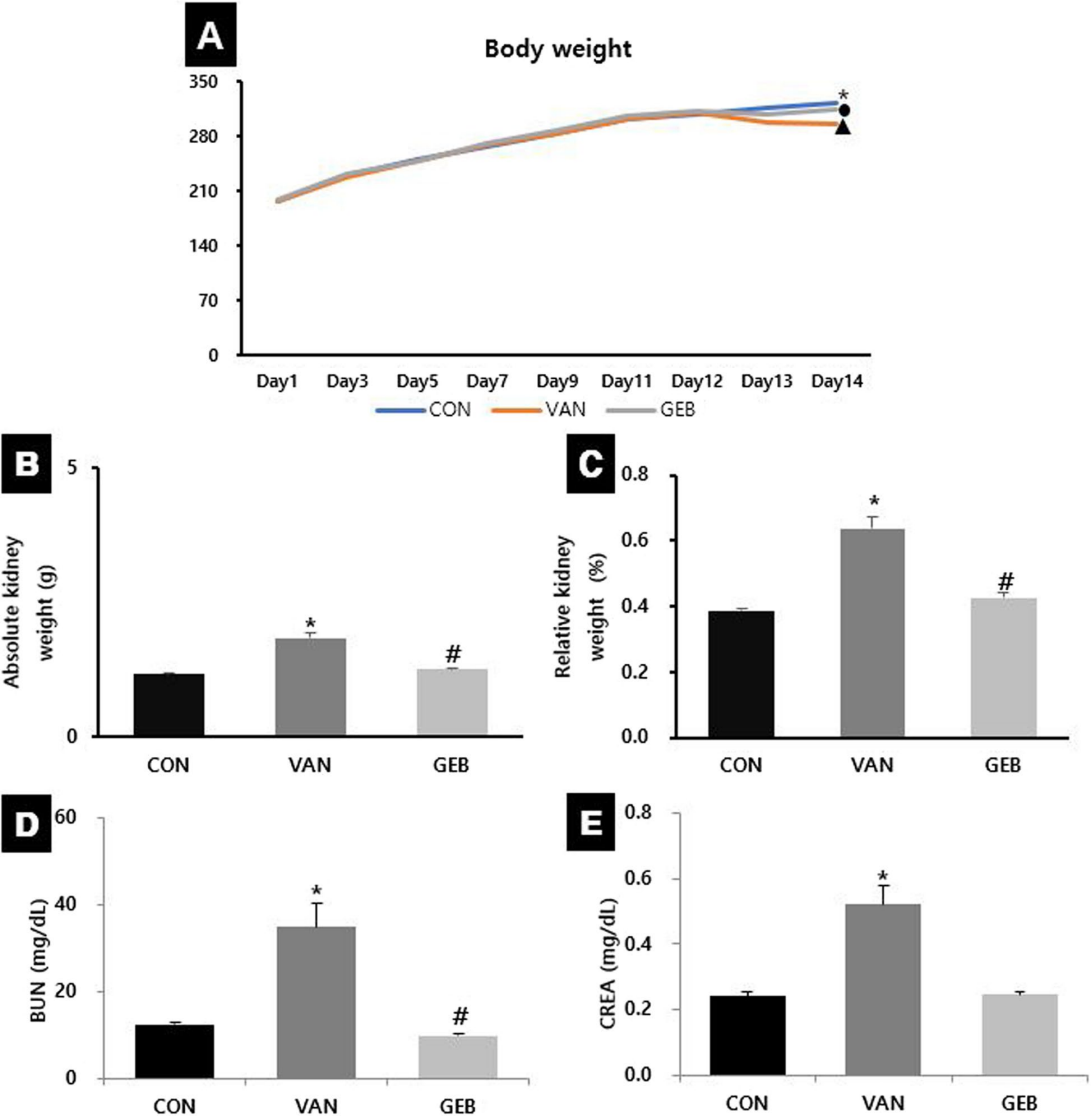
The change of the body weight, the kidney weight, serum BUN and serum CREA. The body weight (**A**) was steadily increased but it was decreased after VAN injection in VAN (circle shape) and GEB group (triangle shape) compared to CON group (*). Absolute kidney weights (**B**) and relative kidney weights (**C**) were significantly increased in the VAN group compared to the CON group and significantly decreased in the GEB group compared to the VAN group. The levels of BUN (**D**) and CREA (**E**) in the serum were significantly increased in the VAN group compared to the CON group and significantly decreased in the GEB group compared to the VAN group. Values were mean ±SE of 8 to 9 rats per group. * vs CON group (*p* < 0.05), # vs VAN group (*p* < 0.01)

The BW of the rats in the CON group gradually increased during the treatment period of 14 days. In the vancomycin administration group, BW tended to decrease on average compared to the CON group after vancomycin administration (Fig. 1A).

### Effects of GEB on absolute and relative weights of kidney

The absolute and relative kidney weights of the VAN group were significantly higher than those of the CON group. In contrast, the absolute and relative kidney weights of the GEB group were significantly lower than those of the VAN group (Fig. 1B, C). The relative weight of kidney was calculated by the following equation:

$$\mathrm{Relative\,weights\,of\,kidney\,}(\mathrm{\%})=\mathrm{Absolute\,weights\,of\,kidney\,}({\text{g}})\,/\mathrm{\,Body\,weight\,}({\text{g}})\times\,100$$

### Effects of GEB on serum levels of BUN and CREA

The serum levels of BUN and CREA in the VAN group were significantly higher than those in the CON group. This indicated that VAN promotes renal damage. The serum levels of BUN and CREA in the GEB group were significantly lower than those in the VAN group (Fig. 1D, E).

### Effect of GEB on histological analysis of the renal section

**Fig. 2:**
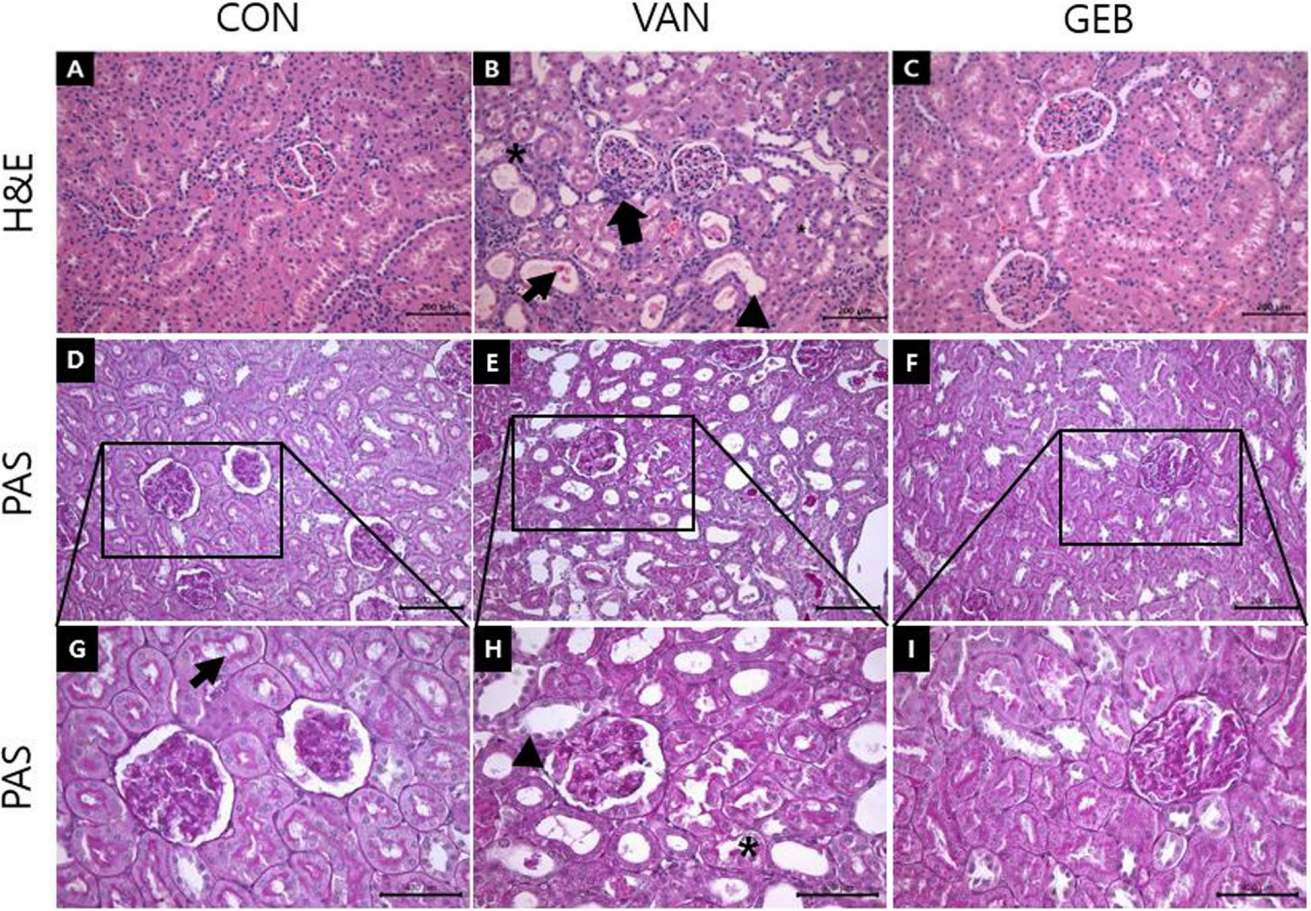
Histopathological changes in H&E and PAS stained kidney tissue. **A**-**C** H&E, **D**-**I** PAS and **A**, **D**, **G** CON group, **B**, **E**, **H** VAN group, **C**, **F**, **I** GEB group. In H&E stained, CON group was showed a normal structure of the kidney. In contrast, the VAN group was showed remarkable damage of kidney, such as tubular dilation (arrow head), tubular cell desquamation (thin arrow), tubular vacuolation (*), interstitial inflammation (arrow). The GEB group was ameliorated these damages than VAN group. In PAS stained, The CON group was showed a normal basement membrane of the tubule and normal brush border (thin arrow) in proximal tubule. In contrast, the VAN group was showed the thinned the basement membrane of the tubule and loss of the brush border (arrow head) in proximal tubule. The GEB group was ameliorated these damages than VAN group. Scale bars in A-F are 200μm, and in G-I 400μm, each

H&E staining revealed that the structures of the glomerulus, proximal tubules, and distal tubule were not affected in the renal section of the CON group (Fig. 2A). In contrast, the renal sections of the VAN group exhibited epithelial detachment, tubular dilation, interstitial edema, and inflammatory cell infiltration (Fig. 2B). The VAN-induced renal damages were ameliorated in the GEB group (Fig. 2C).

The H&E-stained renal sections were scored based on tubular dilation, tubular vacuolation, interstitial inflammation, interstitial edema, and tubular cast. The tubular dilation, tubular vacuolation, interstitial inflammation, interstitial edema, and tubular cast scores in the VAN group were significantly higher than those in the CON group. However, the tubular vacuolation, interstitial inflammation, interstitial edema, and tubular cast scores in the GEB group were significantly lower than those in the VAN group (Table 4).

**Table 4:** Scoring of histological observation in the kidney

	**Tubular dilatation**	**Epithelial cell desquamation**	**Vacuolation**	**Interstitial inflammation**	**Interstitial edema**	**Cast in lumen**
**CON**	0.1±0.06	0.6±0.08	0.2±0.09	0.9±0.08	0.3±0.09	0.1±0.06
**VAN**	1.7±0.17^*^	1.8±0.12^*^	1.5±0.08^*^	1.8±0.20^*^	1.7±0.15^*^	1.4±0.10^*^
**GEB**	0.5±0.05^#^	0.8±0.08^#^	0.4±0.10^#^	1.0±0.05^#^	0.5±0.09^#^	0.4±0.13^#^

^*^vs CON group (*p* < 0.01)

^#^vs VAN group (*p* < 0.01)

PAS staining of the renal sections revealed that the structures of the basement membrane and brush border in the proximal tubules were intact in the CON group (Fig. 2D, G). The proximal tubular basement membrane in the VAN group was not as distinct as that in the CON group. The brush border was barely visible in the VAN group (Fig. 2E, H). The structures of the basement membrane and brush border in the proximal tubules were similar between the GEB and CON groups (Fig. 2F, I).

### Effect of GEB on immunohistochemical analysis of the renal section

**Fig. 3:**
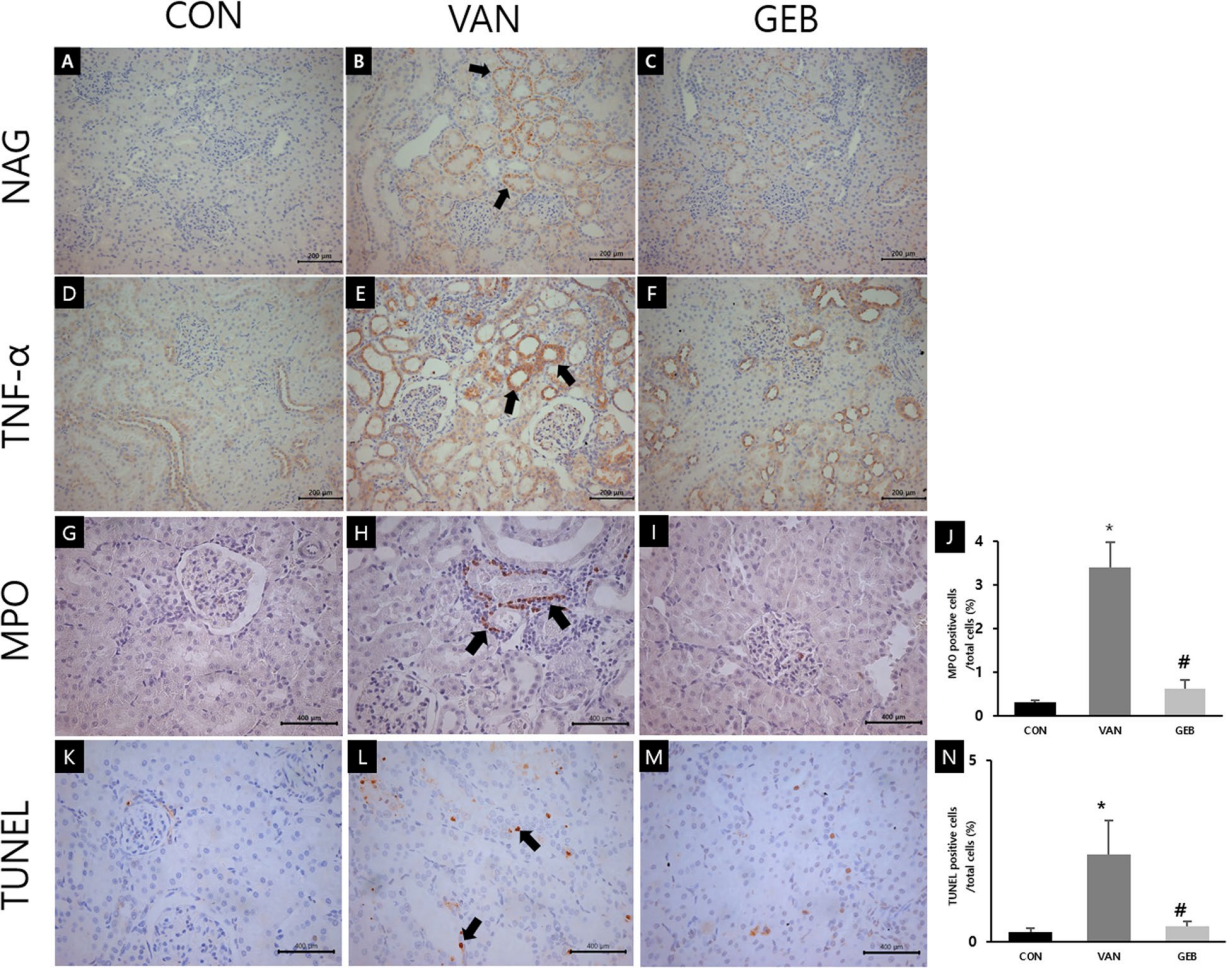
The Results of immunohistochemical of NAG, TNF-α, MPO expression and TUNEL assay. **A**, **B**, **C** NAG, **D**, **E**, **F** TNF-α, **G**, **H**, **I**, **J** MPO. **K**, **L**, **M**, **N** TUNEL and **A**, **D**, **G**, **K** CON group, **B**, **E**, **H**, **L** VAN group, **C**, **F**, **I**, **M** GEB group, **J**, **N** positive cell counting results. As a result of NAG, the CON group was showed little expression of NAG in the cytoplasm of the proximal tubule. In the VAN group, there was strong and more expression of NAG (arrows) compared to CON group. The GEB group was decreased and weaken expression of NAG than VAN group. As a result of TNF-α, the CON group was showed low expression of TNF-α in the cytoplasm of the proximal tubule. In the VAN group, there was strong and more expression of TNF-α (arrows) compared to CON group. The GEB group was decreased and weaken expression of TNF-α than VAN group. As a result of MPO, the VAN group was increased MPO positive cells than CON group. In the GEB group, there was reduced MPO positive cells (arrows) than VAN group. As a result of TUNEL assay, the VAN group was increased TUNEL positive cells compared to the CON group. In the GEB group, there was reduced TUNEL positive cells (arrows) compared to the VAN group. The GEB group was decreased and weaken expression of NAG than VAN group. Scale bars in A-F are 200μm, and in G-M 400μm, each. * vs CON group (*p* < 0.01) # vs VAN group (*p* < 0.01)

NAG, which is expressed in the proximal tubular epithelial lysosome, is released after proximal tubule damage. Immunohistochemical analysis revealed that the expression of NAG in the VAN group (Fig. 3B) was markedly higher than that in the CON group (Fig. 3A). In contrast, the expression of NAG in the GEB group was lower than that in the VAN group (Fig. 3C).

The level of TNF-α, a pro-inflammatory cytokine, in the VAN group (Fig. 3E) was higher than that in the CON group (Fig. 3D). In contrast, the level of TNF-α in the GEB group was lower than that in the VAN group (Fig. 3F).

The expression of MPO, an inflammatory enzyme, is upregulated in the neutrophil granulocytes during inflammation and the neutrophils. The number of MPO-positive cells in the VAN group (Fig. 3H) was significantly higher than that in the CON group (Fig. 3G). Compared with that in the VAN group, the number of MPO-positive cells was significantly lower in the GEB group (Fig. 3I).

TUNEL analysis was performed to examine the effect of GEB on apoptosis of tubular cells. The TUNEL-positive cells were counted and expressed as a percentage (Fig. 3N). The number of TUNEL-positive cells in the VAN group (Fig. 3L) was higher than that in the CON group (Fig. 3K). Compared with that in the VAN group, the number of TUNEL-positive cells was lower in the GEB group (Fig. 3M).

### Effect of GEB on the levels of GSH in the renal tissue

**Fig. 4:**
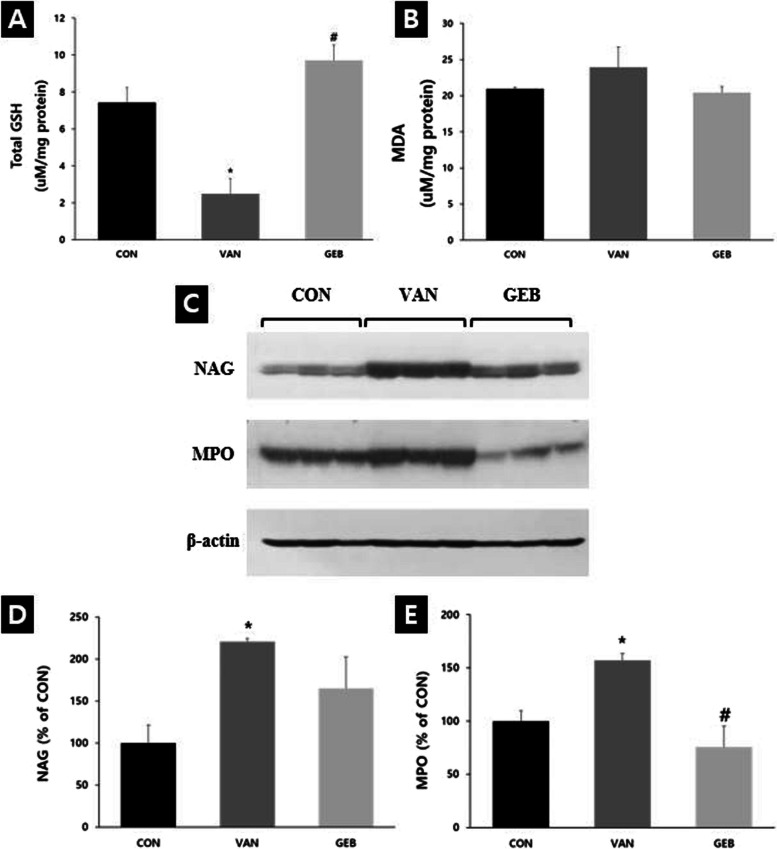
The levels of GSH and MDA and the expressions of NAG and MPO on rats. **A**, **B** GSH and MDA results. GSH is significantly decreased in the VAN group compared to the CON group and significantly increased in the GEB group compared to the VAN group. Values were mean ±SE of 8 to 9 rats per group. MDA is increased in the VAN group compared to the CON group and decreased in the GEB group compared to the VAN group. * vs CON group (*p* < 0.05), # vs VAN group (*p* < 0.01). **C**-**E** Western results. β-actin was used as a loading control. The bar graphs show relative levels of NAG and MPO. Both significantly increased in the VAN group compared to the CON group. NAG expression decreased in the GEB group compared to the VAN group and MPO expression significantly decreased in the GEB group compared to the VAN group. Values were mean ±SE of 3 rats per group. * vs CON group (*p* < 0.01), # vs VAN group (*p* < 0.05)

The levels of total GSH, an antioxidant marker, in the VAN group were significantly lower than those in the CON group. Compared with those in the VAN group, the total GSH levels were significantly higher in the GEB group (Fig. 4A).

The levels of MDA, an oxidative stress marker, in the VAN group were higher than those in the CON group. Compared with those in the VAN group, the MDA levels were higher in the GEB group (Fig. 4B).

### Effect of GEB on western blotting analysis of the renal tissue

NAG is released after renal damage and the expression levels of NAG in the VAN group were significantly higher than those in the CON group. Compared with those in the VAN group, the expression levels of NAG were downregulated in the GEB group (Fig. 4C and D). The expression of MPO is upregulated during inflammation. Compared with those in the control group, the expression levels of MPO were significantly upregulated in the VAN group. The expression levels of MPO in the GEB group were lower than those in the VAN group (Fig. 4C and E).

## Discussion

We investigated the preventive effects of GEB against VAN-induced AKI in rats and the underlying mechanisms. VAN is an antibiotic that inhibits bacterial cell wall biosynthesis by binding to the D-alanyl-D-alanine terminal peptide of the peptidoglycan precursor [16]. Previous studies have reported that VAN is associated with various side effects, including renal injury [5]. The kidney injury induced by VAN is that the injected VAN is mostly eliminated by the kidney, but unremoved VAN accumulates in the kidney, providing for the underlying process of kidney damage [10, 16]. Recent studies have reported that the mechanism underlying renal injury is the induction of oxidative stress [4, 17]. Additionally, VAN-induced ROS production contributes to the pathogenesis of renal injury. ROS promotes lipid peroxidation and consequently disrupts the structure and function of membranes, which results in cellular disorders [9, 17].

GEB, which is a herbal component used in traditional Chinese medicine, is reported to directly scavenge free radicals and downregulate free radical production and lipid peroxidation [12, 18]. HBA is one of the major bioactive components of GEB [12, 19]. Additionally, HBA is reported exhibit anti-apoptosis, antioxidant activity, and downregulate MDA levels [11, 20, 21]. This study investigated the protective effect of GEB extract against VAN-induced AKI in rats.

In a previous study, VAN was administered at a dose of 400 mg/kg BW once a day for 3, 5, 7, and 14 days. The administration of VAN at a dose of 400 mg/kg BW for 3 days resulted in maximum damage to the renal tissue [22]. Therefore, the rats were administered this dose of VAN during the last 3 days of the 14-day experiment period in this study. A recent study demonstrated that GEB extract at a dose of 10 mL/kg BW was effective in preventing liver and kidney injuries [12]. Hence, the rats were orally administered GEB at a dose of 10 mL/kg BW for 14 days in this study.

Significant changes in the BW and kidney weight are important indicators of animal health in the VAN-induced nephrotoxicity experiment [23]. A recent study reported that the administration of VAN significantly decreased the BW due to renal injury, which resulted in anorexia and decreased food intake [24, 25]. In this study, the BW of the VAN and GEB groups, which were injected with VAN during the last 3 days of the 14-day treatment period, was decreased compared to that of the CON group. However, the BW of rats in all treatment groups was not significantly different. The absolute and relative weights of the kidney in the VAN group were significantly higher than those in the CON group. VAN-induced increased kidney weight is associated with kidney injury [24]. In contrast, the absolute and relative weights of the kidney in the GEB group were significantly lower than those in the VAN group. The average higher BW and significantly lower absolute and relative kidney weights observed in the GEB group than in the VAN group may be correlated with the prevention of VAN-induced kidney injury. This indicated that GEB extract prevented VAN-induced kidney injury.

The serum levels of BUN and CREA are biochemical markers of kidney injury [25, 26]. Recent studies have demonstrated that the levels of BUN and CREA are significantly upregulated upon treatment with VAN [26–29]. In this study, the serum levels of BUN and CREA in the VAN group were significantly higher than those in the CON group. Additionally, the serum levels of BUN and CREA in the GEB group were significantly lower than those in the VAN group. This indicated that GEB extract prevents VAN-induced kidney injury through the low serum levels of BUN and CREA.

H&E staining revealed that the renal section of the VAN group exhibited increased tubular dilation, tubular cell desquamation, tubular vacuolation, interstitial inflammation, interstitial edema, and tubular cast. The histopathological scores of the VAN group were significantly higher than those of the CON group. These histological scores can be correlated with the decreased BW and increased absolute and relative weights of the kidney observed in the VAN group. Treatment with GEB prevented the VAN-induced pathological changes in the renal sections. The histopathological scores in the GEB group were significantly lower than those in the VAN group. Additionally, these results are consistent with those of previous histological observations [28]. PAS staining revealed that the renal section of the VAN group did not contain the proximal tubular brush border. This was consistent with the results of other studies, which reported that VAN promotes tubular epithelial desquamation and damages the brush border [22]. VAN is reported to be reabsorbed by the proximal tubule, which results in proximal tubule toxicity [12, 28]. Treatment with GEB mitigated the VAN-induced kidney injury and damage of the brush border in the proximal tubule. This suggested that GEB extract protects against VAN-induced kidney injury and proximal tubular damage.

NAG is a lysosomal enzyme, which is a polymer that does not pass through the glomerular the tubular fluid through the proximal tubule during drug-induced renal injury [30, 31]. The enhanced excretion of NAG can damage the renal proximal tubules [17]. Some studies have reported that the expression of NAG is increased in VAN-induced nephrotoxicity and that the expression of NAG is decreased upon treatment with a therapeutic agent [17]. The changes in NAG levels are consistent with the serum levels of BUN and CREA. The expression of NAG in the VAN group was higher than that in the CON group. Additionally, the expression of NAG in the GEB group was lower than that in the VAN group.

MPO, which is present in the azurophilic granules of neutrophils, generates oxidizing agents [32, 33]. MPO-mediated production of oxidants results in tissue damage through oxidation of biomolecules, including proteins, nucleic acids, lipids, and carbohydrates [32–34]. The expression of MPO in the VAN group was higher than that in the CON group. However, the expression of MPO in the GEB group was lower than that in the VAN group. This observation was consistent with the decreased number of MPO-positive cells and similar to the results of reduction of interstitial inflammation in histological scoring in our study. This suggested that GEB prevents VAN-induced inflammation.

TNF-α, a pro-inflammatory cytokine, plays an important role in epithelial cell damage and apoptosis pathway through the regulation of ROS-activated macrophages [35]. In this study, the expression of TNF-α was assessed using immunohistochemical analysis. The expression of TNF-α in the VAN group was significantly higher than that in the CON group. Additionally, the expression of TNF-α in the GEB group was lower than that in the VAN group. This indicated that GEB alleviates VAN-induced interstitial inflammation by inhibiting the production of pro-inflammatory cytokines. TNF-α also plays an important role in the apoptosis pathway. Apoptosis is upregulated in the renal proximal tubule cells during nephrotoxicity [35, 36]. Hence, the effect of GEB on apoptosis was analyzed using the TUNEL assay. In this study, the number of apoptotic cells in the proximal tubule cells in the VAN group was significantly higher than that in the CON group. However, the number of apoptotic cells in the proximal tubule cells in the GEB group was significantly lower than that in the VAN group. This was consistent with the decreased number of TUNEL-positive cells, which was evaluated using the method of Fan *et al* (2014) [37], in the GEB group. Thus, GEB prevents VAN-induced tubular cell damage by downregulating apoptosis.

Glutathione is an antioxidant that regulates various cellular functions [4, 12]. The reaction between glutathione and ROS generates oxidized glutathione and other disulfides, which function as direct antioxidants [4]. AKI is reported to downregulate the levels of GSH [4, 38]. GEB extract is reported to exhibit antioxidant activity [11]. Therefore, the levels of GSH indicate the degree of VAN-induced kidney injury, as well as the antioxidant effect of GEB extract. In this study, the total GSH levels in the VAN group were significantly lower than those in the CON group. The total GSH levels were similar between the GEB and CON groups. This indicated that GEB extract exerts an antioxidant effect.

To alleviate kidney damage, the production of ROS must be inhibited. Previous studies have reported that GEB inhibits ROS production [18, 39]. ROS production increases the level of MDA, a marker of lipid peroxidation [18]. In this study, the MDA levels increased on average after VAN injection and decreased on average upon pretreatment with GEB. These results suggest the possibility that GEB extracts will protect cells by scavenging ROS.

Additionally, western blotting analysis revealed that the expression levels of NAG and MPO were upregulated in the VAN group and downregulated in the GEB group, which was consistent with the results of immunohistochemical analyses. Thus, GEB extract exerts preventive effects against VAN-induced AKI.

## Conclusions

**Fig. 5:**
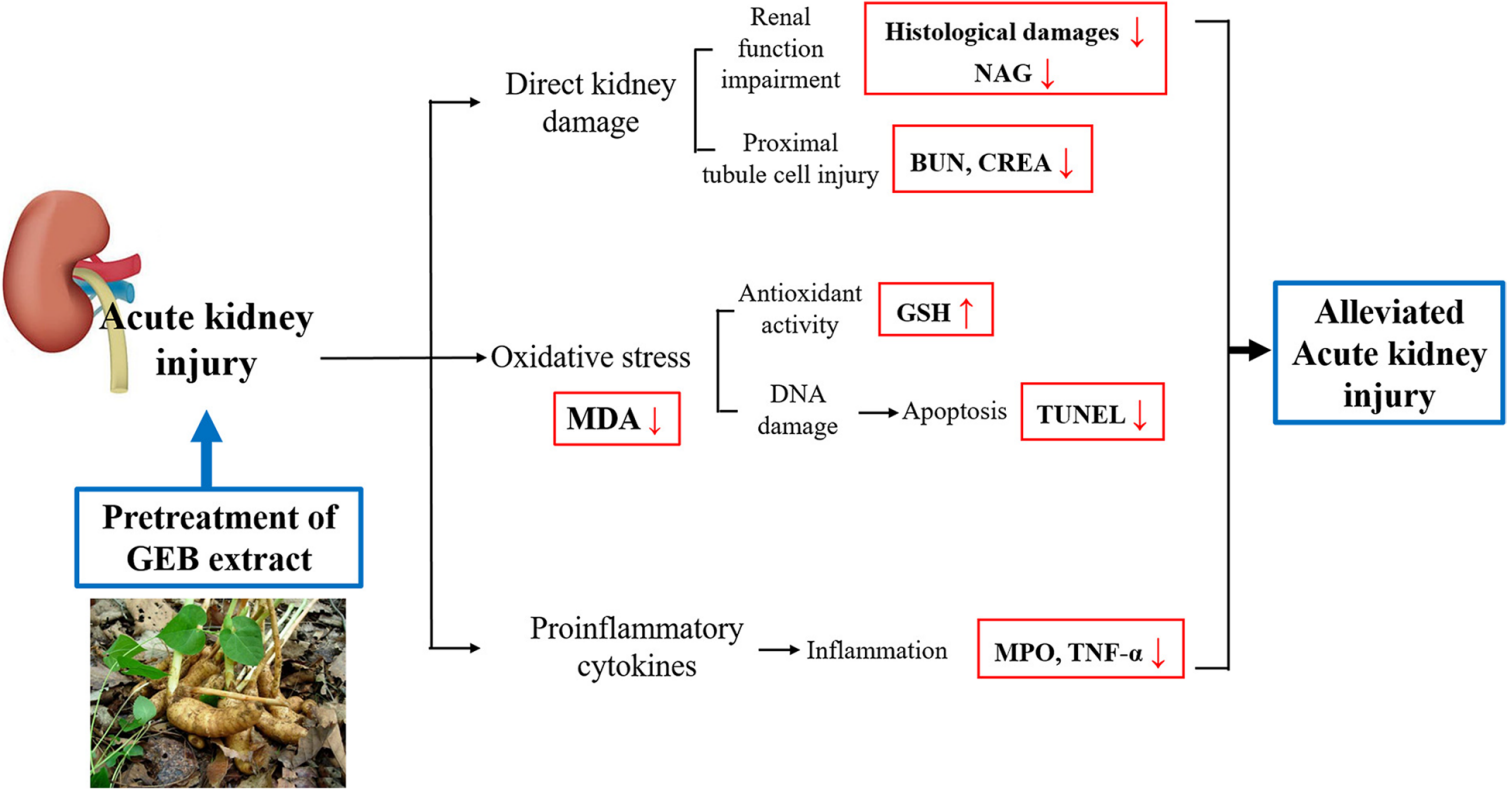
The suggested mechanisms on the preventive effects of *Gastrodia elata* Blume extract

Pretreatment with GEB extract significantly mitigated the VAN-induced enhanced serum BUN and CREA levels and renal damage. GEB extract decreased the expression of inflammatory cytokines and the number of TUNEL-positive cells. Additionally, GEB upregulated the levels of antioxidant enzymes and alleviated oxidative stress (Fig. 5).

In conclusion, this study demonstrated that GEB extract can protect against AKI by exerting anti-inflammatory and anti-oxidative effects.

### Supplementary Information

**Additional file 1: Figure 1.** The chemical structure of vancomycin used in this study. **Figure 2.** Experimental scheme of this study. After adaptation for 7 days, in the CON group was orally administered D.W once a daily for 14 days, and normal saline was i.p. injected for last 3 days after oral administration. In the VAN group, D.W was orally administered and 400 mg/kg of VAN was i.p. injected. In the GEB group, 10 mL/kg of GEB extract was orally administered and VAN was i.p, injected after 1 hour of administration of GEB extract. All rats were sacrificed after 24 hours of last VAN injection.

## Data Availability

The datasets used and analyzed during the current study are available from the corresponding author on reasonable request.

## Abbreviations

AKI: Acute kidney injury
BUN: Blood urea nitrogen
CREA: Creatinine
VAN: Vancomycin hydrochloride
ROS: Reactive oxygen species
NF-κB: Nuclear factor-κB
TNF-α: Tumor necrosis factor-alpha
GEB: Gastrodia elata Blume
HBA: p-hydroxybenzyl alcohol
DW: Distilled water
HPLC: High performance liquid chromatography
NAG: n-acetyl-β-D-glucosaminidase
MPO: Myeloperoxidase
TUNEL: Terminal deoxynucleotidyl transferase dUTP nick end labeling
MDA: Malondialdehyde
BW: Body weight
CON: Control
H&E: Hematoxylin and eosin
PAS: Periodic acid Schiff
DAB: 3,3-diaminobenzidine tetrahydrochloride
POD: Peroxidase
GSH: Total glutathione
