# Supplementary figures and images for: The preventive effect of Gastrodia elata Blume extract on vancomycin-induced acute kidney injury in rats
Source: Lab Anim Res. 2024 Apr 8;40:14. doi: 10.1186/s42826-024-00200-y (PMC11000351; doi:10.1186/s42826-024-00200-y)

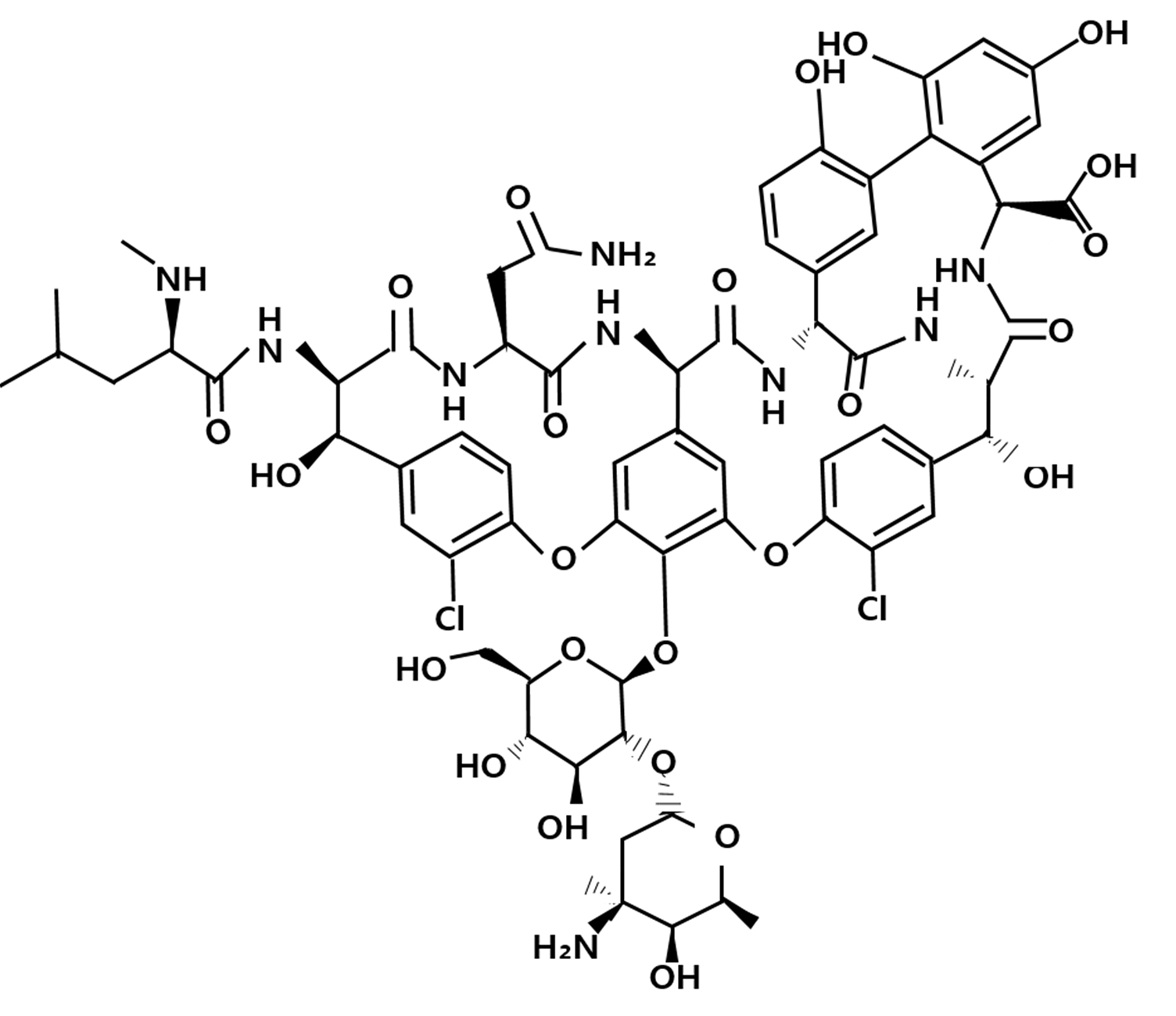

Supplement: Supplementary file 1 — Additional file 1: Figure 1. The chemical structure of vancomycin used in this study. Figure 2. Experimental scheme of this study. After adaptation for 7 days, in the CON group was orally administered D.W once a daily for 14 days, and normal saline was i.p. injected for last 3 days after oral administration. In the VAN group, D.W was orally administered and 400 mg/kg of VAN was i.p. injected. In the GEB group, 10 mL/kg of GEB extract was orally administered and VAN was i.p, injected after 1 hour of administration of GEB extract. All rats were sacrificed after 24 hours of last VAN injection. [file 42826_2024_200_MOESM1_ESM.zip › GEB_supplementary figure/supplementary Figure1.jpg]

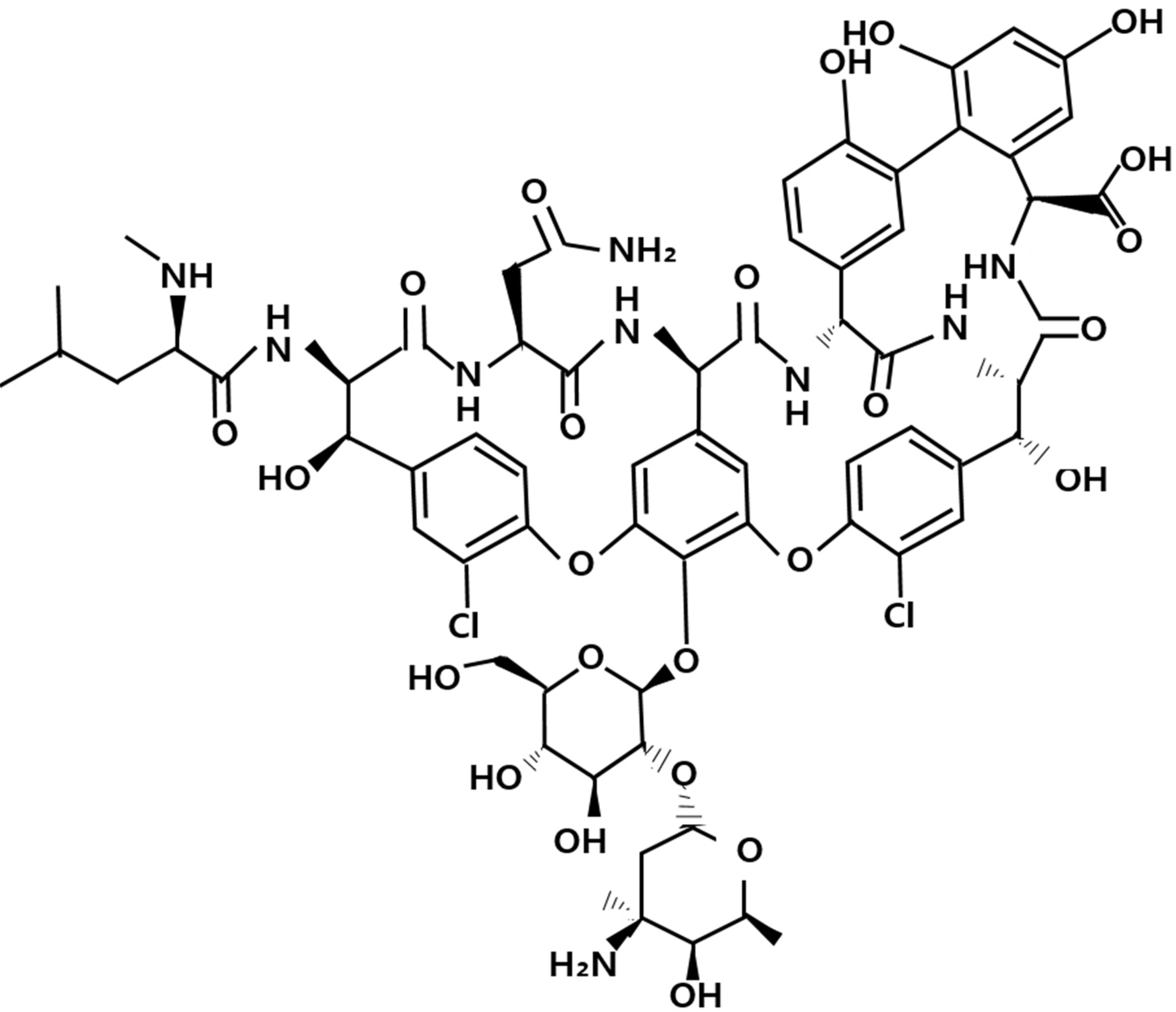

Supplement: Supplementary file 1 — Additional file 1: Figure 1. The chemical structure of vancomycin used in this study. Figure 2. Experimental scheme of this study. After adaptation for 7 days, in the CON group was orally administered D.W once a daily for 14 days, and normal saline was i.p. injected for last 3 days after oral administration. In the VAN group, D.W was orally administered and 400 mg/kg of VAN was i.p. injected. In the GEB group, 10 mL/kg of GEB extract was orally administered and VAN was i.p, injected after 1 hour of administration of GEB extract. All rats were sacrificed after 24 hours of last VAN injection. [file 42826_2024_200_MOESM1_ESM.zip › GEB_supplementary figure/Supplementary figure1.pdf]

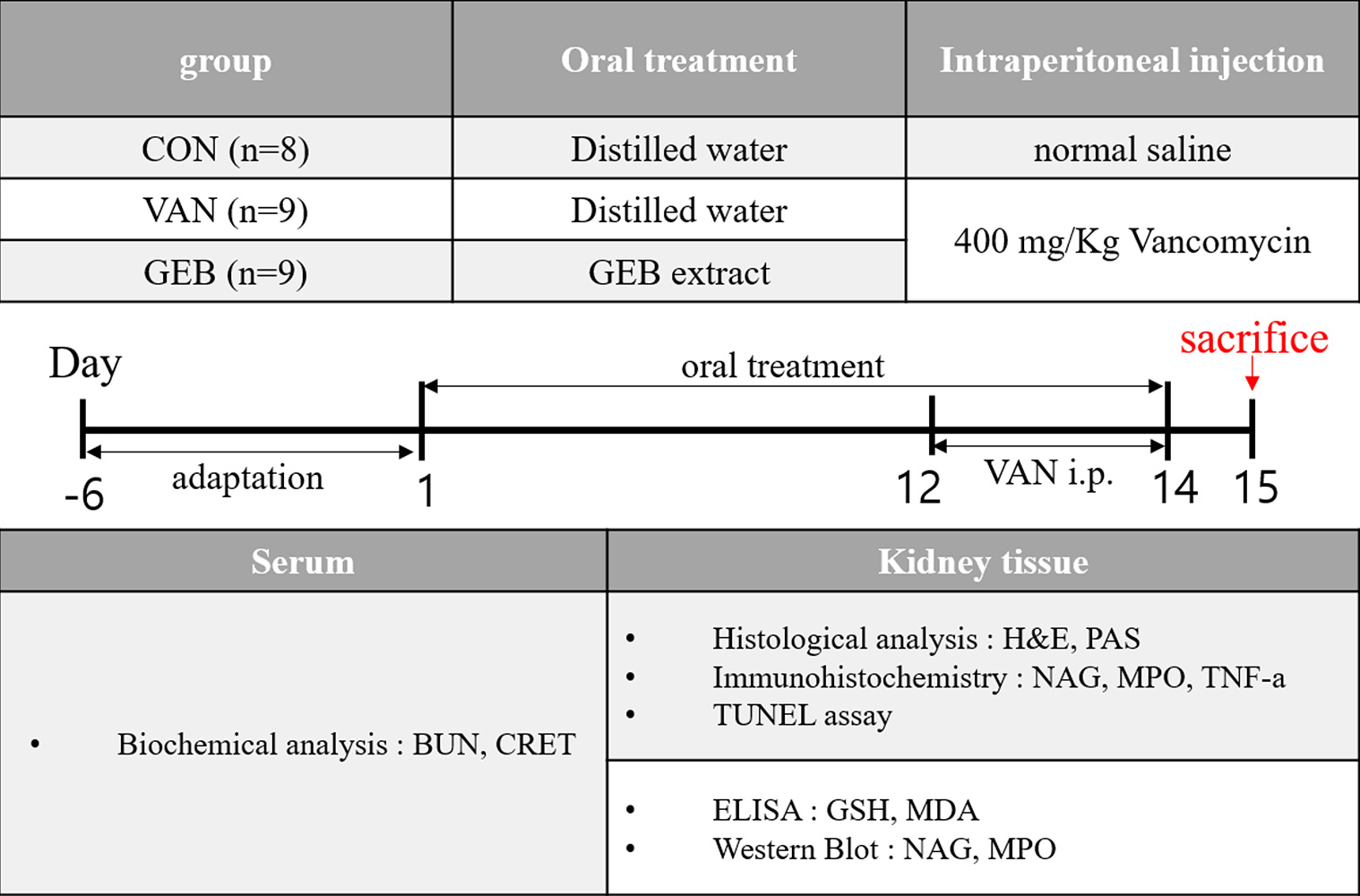

Supplement: Supplementary file 1 — Additional file 1: Figure 1. The chemical structure of vancomycin used in this study. Figure 2. Experimental scheme of this study. After adaptation for 7 days, in the CON group was orally administered D.W once a daily for 14 days, and normal saline was i.p. injected for last 3 days after oral administration. In the VAN group, D.W was orally administered and 400 mg/kg of VAN was i.p. injected. In the GEB group, 10 mL/kg of GEB extract was orally administered and VAN was i.p, injected after 1 hour of administration of GEB extract. All rats were sacrificed after 24 hours of last VAN injection. [file 42826_2024_200_MOESM1_ESM.zip › GEB_supplementary figure/Supplementary figure2.jpg]
